# High loading-dose of dupilumab resulted in rapid disease control in pediatric patients with atopic dermatitis

**DOI:** 10.3389/fimmu.2023.1160710

**Published:** 2023-04-20

**Authors:** Ao Wang, Yuan Zhou, Yang Luo, Yingxia Gao, Jingsi Chen, Wei Li, Xiaoyan Luo, Xu Yao

**Affiliations:** ^1^ Department of Allergy and Rheumatology, Jiangsu Key Laboratory of Molecular Biology for Skin Diseases and STIs, Hospital for Skin Diseases, Institute of Dermatology, Chinese Academy of Medical Sciences and Peking Union Medical College, Nanjing, China; ^2^ Department of Dermatology, Children’s Hospital of Chongqing Medical University, Chongqing, China; ^3^ National Clinical Research Center for Child Health and Disorders, Ministry of Education Key laboratory of Child Development and Disorders, Chongqing, China; ^4^ Department of Dermatology, Huashan Hospital, Fudan University, Shanghai, China

**Keywords:** atopic dermatitis, CCL17/TARC, children, dupilumab, obesity

## Abstract

**Background:**

The real-world experience of dupilumab in Chinese is limited, and the initial loading dose has not yet been deeply explored in patients aged <6 years.

**Objective:**

To explore the efficacy and safety of dupilumab in Chinese patients with moderate-to-severe atopic dermatitis and investigate the effect of higher loading dose for disease control in patients aged <6 years.

**Methods:**

A total of 155 patients were divided into three groups according to age: <6 years, 6-11 years, and >11 years. Among patients aged <6 years, 37 patients received a high loading dose of 300 mg for body weight <15kg or 600 mg for body weight ≥15kg, and another 37 patients received a standard loading dose of 200 mg for body weight <15kg or 300 mg for body weight ≥15kg. Multiple physicians and patient-reported outcome measures were evaluated at baseline and 2, 4, 6, 8, 12, and 16 weeks after dupilumab treatment.

**Results:**

The proportion of patients showing an improvement of ≥75% in the Eczema Area and Severity Index was 68.0% (17/25), 76.9% (10/13), and 62.5% (25/40) in the aged <6, 6-11, and >11 years groups, respectively, at week 16. After increasing the loading dose, 69.6% (16/23) of patients aged <6 years achieved 4-point improvement in Pruritus Numerical Rating Scale at week 2, compared with 23.5% (8/34) of patients receiving standard loading dose (*P* < 0.001). Obesity (odds ratio=0.12, 95% confidence interval: 0.02-0.70) was predictive of a poor response to dupilumab treatment, while female (odds ratio=3.94, 95% confidence interval: 1.26-12.31) predicted good response at week 16. The change of serum C-C motif ligand 17(CCL17/TARC) could reflect the response to dupilumab (*r* = 0.53, *P* = 0.002 in EASI) among patients aged <18 years. No major adverse events were reported during the treatment.

**Conclusions:**

Dupilumab was effective and well-tolerated in Chinese patients with atopic dermatitis. The increased loading dose helped achieve rapid pruritus control in patients aged <6 years.

## Introduction

Atopic dermatitis (AD) is a common inflammatory skin disease that affects approximately 20% of children and 10% of adults worldwide (1, 2). The essential features of AD are recurrent eczematous lesions and intense pruritus, which have a profound negative impact on patient’s quality of life. Although the pathogenesis of AD has not been fully elucidated, immune imbalance, skin barrier dysfunction, and flora disorders are believed to be the critical mechanisms, among which type II immune dysregulation plays a dominant role (1, 2). Interleukin (IL)-4 and IL-13 produced by activated immune cells are the two most critical cytokines in type II inflammation. IL-4 and IL-13 aggravate skin barrier dysfunction and inflammation in patients with AD and promote the production of antigen-specific IgE by B cells. Recent studies have also shown that IL-4 and IL-13 can act directly on neurons and aggravate pruritus in patients with AD (1, 3, 4). Therefore, targeting IL-4 and IL-13 is a potential therapeutic strategy for alleviating the clinical symptoms of AD.

Dupilumab, a monoclonal antibody targeting the shared IL-4 receptor α (IL-4Rα) that inhibits both IL-4 and IL-13, has demonstrated its efficacy and safety in clinical trials and real-world studies (5–12). Currently, it is the first biologics approved for the treatment of moderate-to-severe AD in patients aged ≥6 months (13). However, due to the strict inclusion criteria of clinical trials, the actual conditions of dupilumab in the real world are not reflected in the studies. Although dupilumab has been approved for treatment of moderate to severe pediatric AD patients in China, real-world data from the Chinese pediatric population are still lacking (14–17).

One of the main therapeutic goals for AD is achieving pruritus control as early as possible. The efficacy of dupilumab in relieving pruritus has been verified in several studies (10, 18–20). According to the current recommendation, the therapeutic and loading dose of dupilumab in children depend on their body weight. However, rapid pruritus control in children by increasing the loading dose of dupilumab has not been reported, and the real-world experience of dupilumab treatment in AD patients aged <6 years was also not described. With this background, we reviewed the clinical data of patients who had received dupilumab treatment in the past 2 years. The efficacy and safety of dupilumab treatment in patients aged <6 years was evaluated, as well as those aged ≥6 years. The improvement in pruritus under a high loading dose, serum biomarkers and potential clinical parameters associated with the therapeutic effect of dupilumab were also explored.

## Methods

### Patient characteristics

This was a retrospective study, in which data were collected from the medical records of patients who received dupilumab treatment from July 2020 to January 2023. This study was approved by the Ethics Committee of institute of dermatology, Chinese Academy of Medical Sciences and Peking Union Medical College, and Children’s Hospital of Chongqing Medical University (Ethical approval number: Report No: [2022] Clinic-R [010]). The study has been registered in Chinese Clinical Trial Registry (http://www.chictr.org.cn/index.aspx) under the registration number ChiCTR2200059932.

The clinical data of 180 patients were reviewed, of which 24 patients were excluded because of incomplete clinical information and one patient was excluded because of a diagnosis of other diseases. A total of 155 patients were included in the study. AD was diagnosed using the Hanifin and Rajka criteria (21). Patients were evaluated at baseline, 2, 4, 6, 8, 12, and 16 weeks. A total of 78 patients were followed up for 16 weeks (**Figure S1**).

### Treatment regimen

Fifty-one patients aged >11 years were treated with 300 mg dupilumab Q2W after a loading dose of 600 mg. Thirty patients aged 6-11 years was administrated a maintained dose of 300mg Q2W or Q3W after a loading dose of 600mg. For patients aged <6 years, 37 patients received a high loading dose of 300 mg for body weight <15kg or 600 mg for body weight ≥15kg following by 300mg Q4W (named as high loading dose group in this study). The other 37 patients aged <6 years received a loading dose of 200 mg for body weight <15kg or 300 mg for body weight ≥15kg following by 300mg Q4W (named as standard loading dose group in this study) (8). Detailed treatment regimens are summarized in **Figure S2**.

During dupilumab treatment, the use of topical corticosteroids, calcineurin inhibitors, and moisturizers was still encouraged. Changes in topical corticosteroids were recorded at baseline and 16 weeks.

### Data collection

Data on demographics, body mass index (BMI), disease duration, atopic family history, comorbidities, and specific allergens were collected at baseline. Laboratory tests, including serum total immunoglobulin E (IgE) and eosinophil counts, were recorded at baseline and week 16. Food allergy was self-reported by patients or documented by a physician. Obesity was defined as BMI≥28 kg/m^2^ in adults. For patients aged <18 years, obesity was defined according to the obesity reference in Chinese children (22). Serum total IgE was detected by ImmunoCapTM1000 (Thermo Fisher Scientific, Sweden).

Disease severity was assessed by Scoring Atopic Dermatitis (SCORAD), Eczema Area and Severity Index (EASI), and Investigator’s Global Assessment (IGA). The primary endpoint was the proportion of patients achieving an improvement of ≥75% in EASI (EASI75) or IGA0/1 (or reduction ≥2 from baseline) at week 16. Sleep- Numerical Rating Scale (NRS) and Pruritus-NRS were used to evaluate sleep disturbances and pruritus in the past week.

### Serum collection and biomarkers detection

Serum samples of 34 patients aged <18 years were collected at baseline and after dupilumab treatment. All patients received at least 10 doses of dupilumab. The levels of a panel of 19 biomarkers were measured at baseline and after dupilumab treatment by Luminex technology. The rationale for selection of biomarkers was to cover the major T helper subsets and inflammatory mediators that were related to AD inflammation based on previous publications (23–25).

### Statistical analysis

Continuous variables were reported as mean and standard deviation (SD), and categorical variables as frequencies or percentages. The Wilcoxon paired test was performed to evaluate changes in SCORAD, EASI, IGA, body surface area (BSA), Sleep-NRS, Pruritus-NRS, IgE, eosinophil count, and serum biomarkers over time. The Kruskal-Wallis test and Mann Whitney test was used to compare continuous variables between groups, as appropriate. For the comparison of categorical variables, Fisher’s exact test was used. Pearson’s correlation was computed to analyze the correlation between serum biomarkers and SCORAD, EASI, and Pruritus-NRS. Binary logistic regression was used to assess potential factors associated with the outcome. The outcome was defined as achieving EASI75. *P <*0.05 was considered statistically significant. All data were analyzed using Prism 9.3.1 (GraphPad, San Diego, CA, USA), and SPSS 22.0 (SPSS Inc., Chicago, IL, USA).

## Results

### Demographic data and clinical characteristics of the study population

A total of 155 patients were enrolled in this study. Detailed demographic data and clinical characteristics of all patients were outlined in **Table S1**. Among the 155 patients, 47.7% (74/155) of patients were aged <6 years, 19.4% (30/155) of patients were aged 6–11 years, and 32.9% (51/155) were aged >11 years (**Table 1**). The mean ages of patients in the three groups were 3.4 ± 1.3, 8.5 ± 1.5, and 26.1 ± 15.2 years, respectively. **Table 1** presented a summary of the demographic data and baseline clinical characteristics of the patients.

**Table 1:** Demographic data and clinical characteristics of study population.

Characteristics	<6 years (n=74)	6−11 years (n=30)	> 11 years (n=51)
Sex: Female, n (%)	30 (40.5%)	10 (33.3%)	19 (37.3%)
Ages (year), mean ± SD, (range)	3.4 ± 1.3	8.5 ± 1.5	26.1 ± 15.2
Disease duration (years), mean ± SD	3.1 ± 1.3	7.2 ± 2.2	14.1 ± 9.2
Baseline BMI, mean ± SD Obesity	15.4 ± 1.7 5 (6.8%)	17.1 ± 2.9 4 (13.3%)	22.4 ± 3.8 7 (13.7%)
Baseline SCORAD, mean ± SD > 50, severe, n, % ≤ 50 and > 25, moderate, n, %	61.5 ± 11.0 63 (85.1%) 11 (14.9%)	64.5 ± 16.1 26 (86.7%) 4 (13.3%)	59.3 ± 15.4 36 (70.6%) 15 (29.4%)
Baseline EASI, mean ± SD	21.8 ± 10.6	24.3 ± 13.4	22.4 ± 14.7
Baseline IGA, mean ± SD	3.8 ± 0.4	3.5 ± 0.6	3.5 ± 0.6
Baseline Sleep-NRS, mean ± SD	6.2 ± 2.4	5.1 ± 3.2	4.7 ± 2.7
Baseline Pruritus-NRS, mean ± SD	7.5 ± 1.6	6.8 ± 1.9	6.3 ± 2.0
Baseline BSA, mean ± SD	41.9 ± 22.6	41.2 ± 23.0	44.5 ± 25.6
Baseline IgE, kU/L Available for patients, n, %	828.0 ± 1219.5 62 (83.8%)	2145.3 ± 2012.7 21 (70.0%)	1843.7 ± 1892.9 26 (51.0%)
Specific allergen available for patients, n, % Dust mite allergy, n, %	65 (87.8%) 40 (61.5%)	28 (93.3%) 23 (82.1%)	24 (47.1%) 19 (79.2%)
Baseline eosinophil count (×10^9^/L) Available for patients, n, %	1.0 ± 1.9 62 (83.8%)	1.1 ± 0.8 21 (70.0%)	0.6 ± 0.4 29 (56.9%)
Atopic family history	41 (55.4%)	15 (50.0%)	30 (58.8%)
Allergic comorbidities, n, % Allergic rhinitis, n, % Asthma, n, % Food allergy, n, %	35 (47.3%) 18 (24.3%) 6 (8.1%) 24 (33.3%)	22 (73.3%) 14 (46.7%) 3 (10.0%) 12 (42.9%)	37 (72.5%) 29 (56.9%) 9 (17.6%) 15 (34.9%)
Treatment before the use of dupilumab Topical corticosteroids Topical calcineurin inhibitors Topical phosphodiesterase 4 inhibitor Systemic corticosteroids Oral antihistamines Leukotrienes receptor antagonist Immunosuppressants	64 (86.5%) 45 (60.8%) 5 (6.8%) 5 (6.8%) 41 (55.4%) 0 0	26 (86.7%) 13 (43.3%) 0 4 (13.3%) 27 (90.0%) 1 (3.3%) 1 (3.3%)	41 (80.4%) 29 (56.9%) 0 7 (13.7%) 42 (82.4%) 5 (9.8%) 6 (11.8%)

BSA, body surface area; EASI, Eczema Area and Severity Index; IGA, Investigator’s Global Assessment; IgE, immunoglobulin E; NRS, Numerical Rating Scale; SCORAD, Scoring Atopic Dermatitis; SD, standard deviation.

Food allergy was available for 72 patients aged <6 years, 28 patients aged 6-11 years, and 43 patients aged >11 years.

The baseline clinical features of the groups were not identical. The proportion of obesity was 6.8% (5/74) among patients aged <6 years, 13.3% (4/30) among those aged 6–11 years, and 13.7% (7/51) among those aged >11 years (**Table 1**). Dust mites were the most common allergens in all patients. The percentage of patients with atopic family history was similar among all groups. However, the proportion of patients with allergic comorbidities was 47.3% (35/74) among those aged <6 years, which was lower than the other two groups (73.3% [22/30] in patients aged 6–11 years and 72.5% [37/51] in patients aged >11 years; **Table 1**). Allergic rhinitis was the most common concomitant disease in patients aged 6–11 years and >11 years, reported by 46.7% (14/30) and 56.9% (29/51), respectively, and it was 24.3% (18/74) in patients aged <6 years (**Table 1**). Food allergy was the most often allergic comorbidities in patients aged <6 years, reported by 33.3% (24/72), while it was 42.9% (12/28) and 34.9% (15/43) in patients aged 6–11 years and >11 years, respectively (**Table 1**). The prevalence of asthma was lower compared with allergic rhinitis and food allergy, reported by 8.1% (6/74), 10.0% (3/30) and 17.6% (9/51) in patients aged <6 years, 6–11 years and >11 year, respectively (**Table 1**).

Prior to dupilumab treatment, all patients received multiple medications. Oral antihistamines, topical corticosteroids, and calcineurin inhibitors were the most frequently used drugs. Topical phosphodiesterase 4 inhibitor, systemic corticosteroids, immunosuppressants, and oral leukotriene receptor antagonists were also used in some patients. Details of drug use were summarized in **Tables 1** and **S1**.

### Efficacy outcomes after dupilumab treatment at week 16

**Figure 1:**
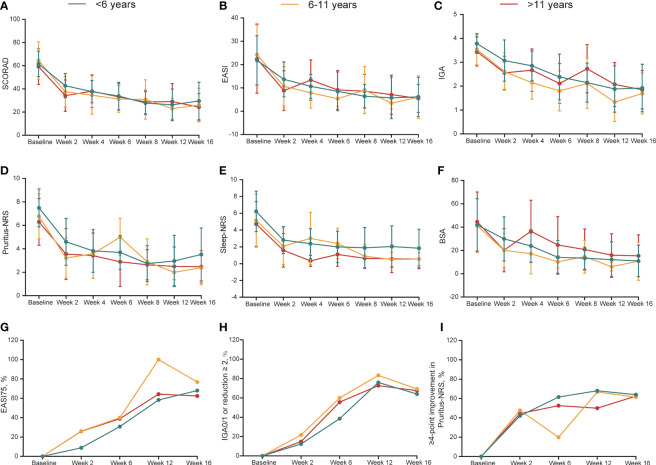
**(A-F)** Dynamic change in SCORAD, EASI, IGA, Pruritus-NRS, Sleep-NRS and BSA during treatment duration across different groups. Data are shown with mean ± SD. **(G-I)** Dynamic change in the percentage of EASI75, IGA0/1 or reduction ≥2 from baseline, ≥4-point improvement in Pruritus-NRS during treatment duration. BSA, body surface area; EASI, Eczema Area and Severity Index; EASI75, at least 75% improvement from baseline in EASI; IGA, Investigator’s Global Assessment; NRS, Numerical Rating Scale; SCORAD, Scoring Atopic Dermatitis; SD, standard deviation.

A fast, continuous, and stable improvement was observed during dupilumab treatment (**Figures 1A–F**, **S3A-F**). In patients aged <6 years, the mean scores of SCORAD, EASI, Pruritus-NRS, Sleep-NRS, and BSA decreased by 52.8%, 72.5%, 49.4%, 68.4%, and 69.3%, respectively, from baseline to week 16 (**Table 2**). In patients aged 6–11 years, corresponding reduction in SCORAD, EASI, Pruritus-NRS, Sleep-NRS, and BSA was 55.9%, 62.4%, 57.2%, 74.6%, and 55.7%, respectively, at week 16 (**Table 2**). For patients aged >11 years, the decreases were 58.3%, 69.5%, 58.5%, 87.0%, and 61.9%, respectively, at week 16 (**Table 2**).

**Table 2:** Outcomes after dupilumab treatment at week 16.

Outcome	<6 years (n= 25)	6−11 years (n=13)	>11years (n=40)	*P value*
EASI50 at week 16, n, %	20 (80.0%)	10 (76.9%)	34 (85.0%)	0.79
EASI75 at week 16, n, %	17 (68.0%)	10 (76.9%)	25 (62.5%)	0.65
EASI90 at week 16, n, %	7 (28.0%)	6 (46.2%)	11 (27.5%)	0.41
IGA0/1 or reduction ≥ 2-point at week 16, n, %	16 (64.0%)	9 (69.2%)	27 (67.5%)	0.95
Proportion of ≥4-point improvement in Pruritus-NRS from baseline to week 16, n, %	16 (64.0%)	8 (61.5%)	25 (62.5%)	1.00
SCORAD at week 16, mean ± SD	30.0 ± 16.9^†^	26.1 ± 14.5^†^	24.3 ± 11.6^†^	N/A
Mean percent change in SCORAD from baseline to week 16, mean ± SD	−52.8 ± 26.7	−55.9 ± 27.4	−58.3 ± 18.1	0.84
EASI score at week 16, mean ± SD	6.2 ± 8.4^†^	6.1 ± 9.1^§^	5.5 ± 6.0^†^	N/A
Mean percent change in EASI from baseline to week 16, mean ± SD	−72.5 ± 28.9	−62.4 ± 61.9	−69.5 ± 33.4	0.59
Pruritus-NRS score at week 16, mean ± SD	3.5 ± 2.3^†^	2.4 ± 1.4^†^	2.5 ± 1.4^†^	N/A
Mean percent change in Pruritus-NRS from baseline to week 16, mean ± SD	−49.4 ± 34.0	−57.2 ± 32.9	−58.5 ± 26.2	0.55
Sleep-NRS score at week 16, mean ± SD	1.8 ± 2.3^†^	0.5 ± 0.8^§^	0.6 ± 1.2^†^	N/A
Mean percent change in Sleep-NRS from baseline to week 16, mean ± SD	−68.4 ± 42.7	−74.6 ± 40.3	−87.0 ± 25.6	0.03
Percentage of BSA at week 16, mean ± SD	10.9 ± 13.6^†^	10.8 ± 16.5^§^	15.3 ± 18.0^†^	N/A
Mean percent change in the percentage of BSA from baseline to week 16, mean ± SD	−69.3 ± 31.1	−55.7 ± 78.1	−61.9 ± 38.5	0.21

BSA, body surface area; EASI, Eczema Area and Severity Index; EASI50, at least 50% improvement from baseline in EASI; EASI-75, at least 75% improvement from baseline in EASI; EASI-90, at least 90% improvement from baseline in EASI; IGA, Investigator’s Global Assessment; N/A: not applicable; NRS, Numerical Rating Scale; SCORAD, Scoring Atopic Dermatitis; SD, standard deviation.

^†^P < 0.001 versus baseline, ^§^P < 0.01 versus baseline.

Comparisons among different age groups.

Seventy-eight patients were followed up at week 16. Patients of different ages responded well to dupilumab treatment. The rates of EASI75 in patients aged <6 years and 6–11 years were 68.0% (17/25) and 76.9% (10/13), respectively, at week 16, while it was 62.5% (25/40) in patients aged >11 years (**Table 2**, **Figure 1G**). Meanwhile, 28.0% (7/25) and 46.2% (6/13) of patients aged <6 and 6–11 years, respectively, achieved EASI90 at week 16 compared with 27.5% (11/40) of patients aged >11 years (**Table 2**). After 16 weeks of treatment, the proportion of IGA0/1 (or reduction ≥2 from baseline) among the patients aged <6, 6–11, and >11 years was 64.0% (16/25), 69.2% (9/13), and 67.5% (27/40), respectively (**Table 2**, **Figure 1H**). Pruritus was also significantly relieved after dupilumab treatment at week 16. The proportion of patients with ≥4-point improvement in Pruritus-NRS was 64.0% (16/25), 61.5% (8/13), and 62.5% (25/40) among patients aged <6, 6–11, and >11 years (**Table 2**, **Figure 1I**). Collectively, these data showed that dupilumab treatment was effective among the different age groups.

### Rapid disease control after increasing the loading dose of dupilumab in patients aged <6 years

**Figure 2:**
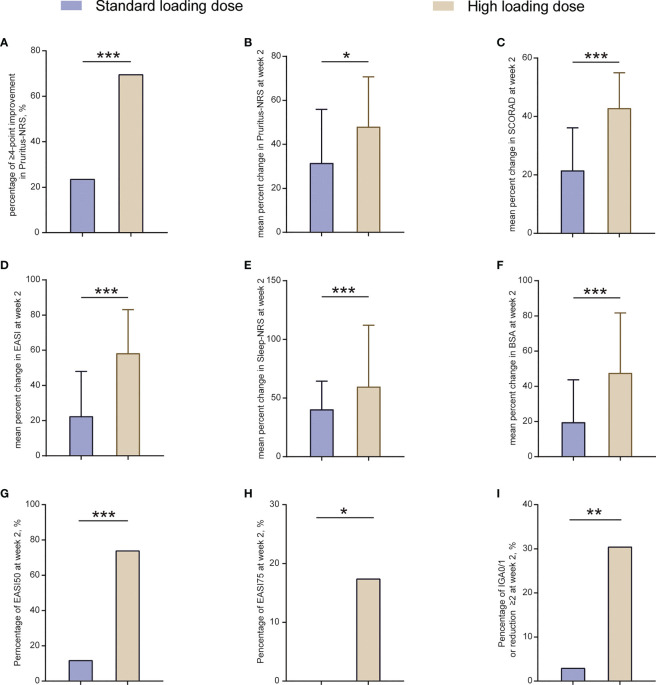
Outcomes at week 2 between patients receiving standard loading dose and high loading dose group. BSA, body surface area; EASI, Eczema Area and Severity Index; EASI50, at least 50% improvement from baseline in EASI; EASI-75, at least 75% improvement from baseline in EASI; IGA, Investigator’s Global Assessment; NRS, Numerical Rating Scale; SCORAD, Scoring Atopic Dermatitis. *P < 0.05, **P < 0.01, ***P < 0.001.

A total of 50.0% (37/74) of patients aged <6 years were administrated with a high loading dose in our study; therefore, we compared the initial efficacy with patients receiving a standard loading dose. Baseline clinical features of these two groups was shown in **Table S2**. Although there were some differences in baseline clinical features, the disease severity, EASI score, and pruritus scores was comparable. We found that at week 2, more patients achieving ≥4-point improvement in Pruritus-NRS in the high loading dose group, compared with that in the standard loading dose group (69.6% [16/23] vs. 23.5% [8/34], *P <*0.001; **Figure 2A**), which was in line with the decrease of Pruritus-NRS (48.0% vs. 31.5%, *P* = 0.01; **Figure 2B**).

Additionally, a more remarkable decrease in percent change of SCORAD, EASI, Sleep-NRS, and BSA was observed in the high loading dose group at week 2, compared with that in the standard loading dose group (42.8% vs. 21.5% of SCORAD, *P <*0.001; 58.3% vs. 22.5% of EASI, *P <*0.001; 59.7% vs. 40.3% of Sleep-NRS, *P <*0.001; 47.6% vs. 19.6% of BSA, *P <*0.001; **Figures 2C–F**). Moreover, the proportion of EASI50, EASI75, and IGA0/1 (or reduction ≥2 from baseline) in the high loading dose group was also significantly higher than that in the standard loading dose group (73.9% [17/23] vs. 11.8% [4/34] of EASI50, *P <*0.001; 17.4% [4/23] vs. 0 [0/34] of EASI75, *P* = 0.02; 30.4% [7/23] vs. 2.9% [1/34] of IGA0/1 [or reduction ≥2 from baseline], *P* = 0.005; **Figures 2G–I**). Overall, this outcome indicated that the high loading dose helped in achieving rapid symptom control in patients aged <6 years.

At week 16, some numerical differences were noted in the percentage of improvement between the groups, but the differences were not statistically significant (**Figure S4**, **Table S3**).

### Correlation of clinical parameters with the efficacy of dupilumab treatment

**Figure 3:**
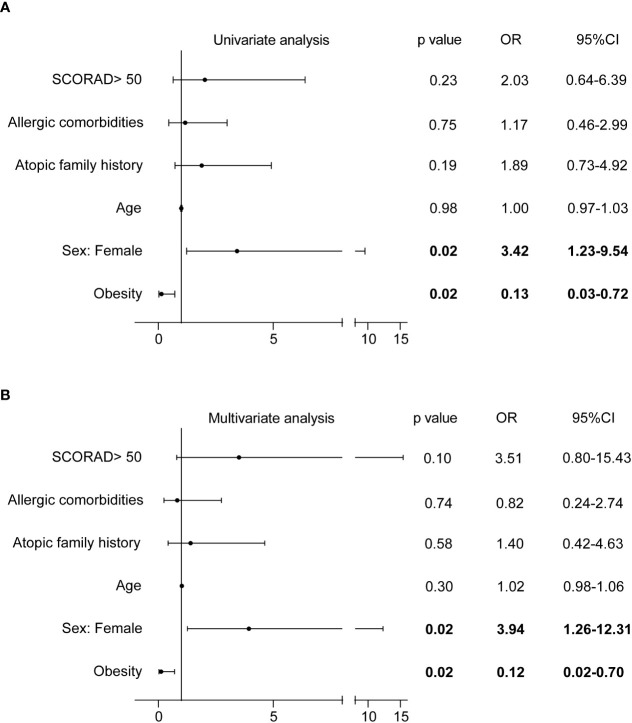
Correlation of clinical parameters with EASI75 at week 16. CI, confidence interval; EASI, Eczema Area and Severity Index; EASI-75, at least 75% improvement from baseline in EASI; OR, odds ratio; SCORAD, Scoring Atopic Dermatitis.

At week 16, 66.7% (52/78) of patients achieved EASI75, whereas the remaining 33.3% (26/78) did not (**Figure S3G**). Logical regression analysis was applied to explore potential clinical parameters affecting the response to dupilumab, including age, sex, obesity, atopic family history, allergic comorbidities, and disease severity (SCORAD > 50). The proportion of obese patients who achieved EASI75 at week 16 was significantly lower than that of non-obese patients (25.0% [2/8] vs.71.4% [50/70], *P* = 0.01; **Figure S5A**), and more female patients achieved EASI75 at week 16 compared with male patients (80.6% [29/36] vs.54.8% [23/42], *P* = 0.02; **Figure S5B**). The relationship between obesity and poor response to dupilumab was confirmed by logical regression analysis (odds ratio [OR] = 0.13, 95% confidence interval [CI]: 0.03-0.72 in univariate analysis, and OR = 0.12, 95%CI: 0.02–0.70 in multivariate analysis; **Figure 3**). In addition, females were also identified as an independent factor for good response to dupilumab (OR=3.42, 95%CI: 1.23-9.54 in univariate analysis, and OR = 3.94, 95%CI: 1.26–12.31 in multivariate analysis; **Figure 3**). The therapeutic effect of dupilumab at week 16 was not correlated with age, atopic family history, allergic comorbidities, or disease severity (**Figure 3**).

### Changes in laboratory tests and biomarkers after dupilumab treatment

At week 16, the serum total IgE level reduced significantly compared with the baseline (1324.1 ± 1660.4 vs. 561.5 ± 991.2, *P <*0.001; **Figure S6A**), whereas no significant decline was noted in the eosinophil count (0.8 ± 0.6×10^9^ vs. 0.8 ± 0.9×10^9^, *P* = 0.12; **Figure S6B**).

**Figure 4:**
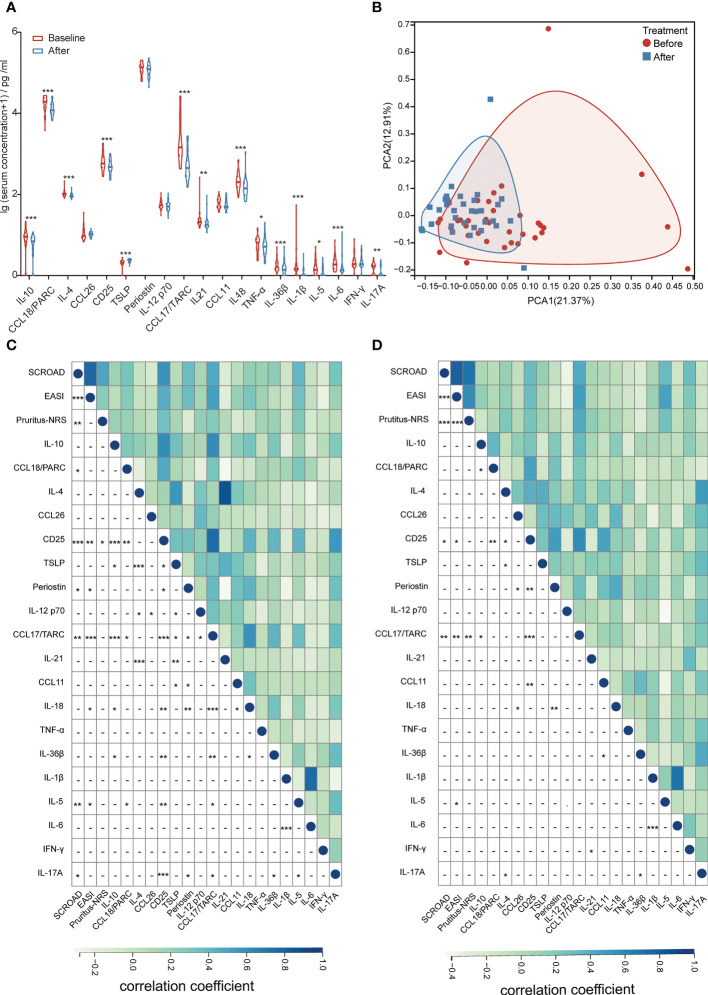
**(A)** Changes in the levels of serum biomarkers at baseline and after dupilumab treatment. **(B)** PCA analysis of patients before and after dupilumab treatment. **(C)** Correlation between clinical scores and serum biomarkers at baseline. **(D)** Correlation between the change in clinical scores and serum biomarkers after dupilumab treatment. CCL, C-C motif ligand; EASI, Eczema Area and Severity Index; IFN, interferon; IL, interleukin; NRS, Numerical Rating Scale; PARC, pulmonary and activation-regulated chemokine; PCA, principal component analysis; SCORAD, Scoring Atopic Dermatitis; TARC, thymus and activation-regulated chemokine; TNF, tumor necrosis factor; TSLP, thymic stromal lymphopoietin. *P < 0.05, **P < 0.01, ***P < 0.001.

Serum biomarkers related to AD were detected in 34 patients aged <18 years. After dupilumab treatment, levels of most biomarkers, including IL-10, C-C motif ligand (CCL) 18/PARC, IL-4, CD25, CCL17/TARC, IL-21, IL-18, tumor necrosis factor -α, IL-36β, IL-1β, IL-5, IL-6, and IL-17A were reduced, whereas the level of thymic stromal lymphopoietin was elevated compared with baseline (**Figure 4A**, **Table S4**). The level of other biomarkers, including CCL26, periostin, IL-12p70, CCL11, and interferon-γ did not change after dupilumab treatment (**Figure 4A**, **Table S4**). Although the levels of most biomarkers had dramatically changed after treatment, principal component analysis (PCA) did not show an obvious distinction (**Figure 4B**).

Correlation analysis between serum biomarkers and clinical scores was conducted to identify potential biomarkers that could reflect disease severity and therapeutic response. We found that the levels of CCL18/PARC, CD25, periostin, CCL17/TARC, IL-18, IL-5, and IL-17A at baseline reflected the disease severity (**Figure 4C**). After comparing the changes in clinical scores, we found that the decrease in CCL17/TARC, CD25, and IL-5 was positively related to the improvement of SCORAD, EASI, and (or) Pruritus-NRS, particularly CCL17/TARC, which showed the strongest correlation with the change of clinical scores (*r* = 0.47, *P* = 0.006 with SCORAD; *r* = 0.53, *P* = 0.002 with EASI; *r* = 0.47, *P* = 0.006 with Pruritus-NRS; **Figure 4D**).

### Safety and adjustment of topical corticosteroids after dupilumab treatment

Adverse events (AEs) were reported by 21.3%(33/155) of patients in this study (**Table 3**). Conjunctivitis was the most common AEs, noted in 19 patients 12.3%. Facial erythema was reported in 8 patients (5.2%). Other AEs included injection-site reactions (2.6%, 4/155), flu-like symptoms (1.3%, 2/155), joint pain (0.6%, 1/155), herpes simplex virus infection (0.6%, 1/155), and drowsiness (0.6%, 1/155). All AEs were mild and did not lead to treatment discontinuation. Additionally, the incidence of AEs in high loading dose group did not increase compared with that in the standard loading dose group (**Table S5**).

**Table 3:** Adverse events during dupilumab treatment.

Adverse events	<6 years (n=74)	6−11 years (n=30)	>11 years (n=51)	Total (n=155)
Conjunctivitis or aggravation of conjunctivitis	8 (10.8%)	6 (20.0%)	5 (9.8%)	19 (12.3%)
Facial erythema	3 (4.1%)	2 (6.7%)	3 (5.9%)	8 (5.2%)
Injection-site reaction	0	1 (3.3%)	3 (5.9%)	4 (2.6%)
Flu-like symptoms	2 (2.7%)	0	0	2 (1.3%)
Joint pain	0	0	1 (2.0%)	1 (0.6%)
Herpes simplex virus infection	1 (1.4%)	0	0	1 (0.6%)
Drowsiness	1 (1.4%)	0	0	1 (0.6%)

Dupilumab also lessened the use of topical corticosteroids. At week 16, the proportion of patients using high-potency corticosteroids reduced from 48.4% (75/155) to 12.8% (10/78) (*P* < 0.001; **Table S6**).

## Discussion

To the best of our knowledge, this study is the first real-world research that enrolled pediatric patients in China, including those aged <6 years, to study the efficacy and safety of dupilumab in treating AD. Our data showed that dupilumab was effective and well-tolerated in Chinese children with moderate-to-severe AD, and the increased loading dose resulted in rapid pruritus control. Furthermore, we found that obesity and females was associated with response to dupilumab treatment; CCL17/TARC showed the strongest correlation with the improvement in clinical scores among patients aged <18 years.

It has been widely acknowledged that AD was far beyond a skin disorder. Due to the driving of type II inflammation, the prevalence of allergic comorbidities was significantly elevated in individuals with AD, and some patients experienced AD in infancy, and gradually developed into allergic asthma and allergic rhinitis in childhood, which was called atopic march (26, 27). Recent meta-analysis reported that the overall pooled prevalence of allergic rhinitis, asthma and food allergy in AD patients was 40.5%, 25.7% and 32.7%, respectively, which was significantly higher than patients without AD (28–30). And food allergy was more common in children compared with adults (30). The overall prevalence of allergic rhinitis and food allergy in our data was comparable with the published data, but the prevalence of asthma was lower, which may be attributed to the small sample size. Dupilumab was proven to be an efficacious inhibitor of type II inflammation by blocking IL-4Rα, thus, it could alleviate the symptoms of AD, allergic rhinitis, and asthma at the same time (31). A recent study also endorsed that the dupilumab provided a possible additional advantage for AD patients with concomitant food allergy (32). Additionally, whether early intervention of dupilumab could block the progress of atopic march is also an exciting issue and need more research to confirm.

Achieving early pruritus control is an important goal in AD treatment. In this study, we observed that more than two-thirds of patients aged <6 years achieved ≥4-point improvement in Pruritus-NRS at week 2 after increasing the loading dose, which was significantly higher than that reported in the standard loading dose group and previous clinical trial (8). Besides, more patients achieved EASI50 and EASI75 at week 2. Although patients aged <6 years were treated with a high loading dose, the incidence of AEs did not increase during the treatment. Conjunctivitis and facial erythema were the most common AEs, consistent with previous studies (8, 14–17). Our study suggested that dupilumab was a safe treatment for children, and a high loading dose could help to achieve rapid pruritus control.

Previous clinical studies showed an EASI75 response rate of 69.7% and 67.2% at week 16 in patients aged 6–11 years (33). As for patients aged <6 years, 53% achieved EASI75 at week 16 in a recent clinical trial (8). Compared with these clinical studies, a higher response of EASI75 was reported in patients aged <6 years and 6–11 years at week 16 in our study, especially those aged 6–11 years. The higher efficacy in our study may be because the use of combined medications, such as high- and very-high-potency topical corticosteroids, was allowed during treatment. The EASI75 response rate in patients aged >11 years of this study was comparable to the 42–84.6% rate reported in previous real-world studies in China (14–16).

Weight, sex, age, clinical phenotypes, and serum lactate dehydrogenase levels might affect the efficacy of dupilumab treatment (14, 34, 35). Our study also highlighted that obesity predicted poor treatment outcomes, and females responded better to dupilumab treatment, which was in line with the findings of another study in China (14). They found that both BMI <24 kg/m^2^ and females were associated with a better response and suggested that older patients (patients aged ≥ 60 years) tended to respond poorly to dupilumab. Our data could not support this conclusion, which may be related to the low number of older patients included in our study (n = 2). The negative impact of obesity on dupilumab treatment may be related to the low drug distribution in obese patients. However, studies have also shown that elevated serum leptin in obese patients contributed to the proliferation of T-helper (Th) 2 cells and release of IL-4 and 13 (36). Another study showed that obesity induced Th17, rather than Th2, immune response in AD model (37). Collectively, the immune response of obese patients with AD differed from that of non-obese patients with AD. Further studies are needed to optimize the clinical application of dupilumab in obese patients.

As a chemoattractant for Th2 cells, CCL17/TARC has played a key role in the pathogenesis of AD (38). The robust correlation of CCL17/TARC with the severity of AD and therapeutic response has been confirmed in several studies across different ages (23). In our study, CCL17/TARC also showed the strongest correlation with improvement in the clinical scores in patients aged <18 years, compared with IL-5 and CD25, which were also associated with the improvement in EASI and SCORAD. Thus, CCL17/TARC could be used to evaluate the response to dupilumab treatment. In addition, although the levels of most serum cytokines and chemokines decreased significantly after dupilumab treatment, the immune features of patients were not markedly distinctive on PCA. This indicated that the immune imbalance of AD may persist constantly, which may account for AD relapse after dupilumab discontinuation.

Our study has some potential limitations. First, this study was retrospective, and many patients were lost to follow-up. Due to the limited sample size, it was challenging to analyze potential factors affecting the therapeutic effect of dupilumab in different age groups. Second, the follow-up time was only 16 weeks, so it was not easy to evaluate the maintenance after the discontinuation of dupilumab and the safety of long-term treatment.

In conclusion, our study verified the efficacy and safety of dupilumab in Chinese patients with AD. The increased loading doses were safe in patients aged <6 years and helped achieve rapid pruritus control. Sex and obesity may predict the response to dupilumab. CCL17/TARC was found to be a useful serum biomarker for monitoring treatment efficacy in patients aged <18 years. Based on our findings, for the purpose to achieve rapid disease control, we kindly recommend the application of high loading dose in patients aged <6 years, and more clinical trials are necessary to explore the application of dupilumab with high loading dose.

## Data availability statement

The original contributions presented in the study are included in the article/**Supplementary Material**. Further inquiries can be directed to the corresponding authors.

## Ethics statement

The studies involving human participants were reviewed and approved by Ethics Committee of Institute of Dermatology, Chinese Academy of Medical Sciences and Peking Union Medical College. Written informed consent to participate in this study was provided by the participants’ legal guardian/next of kin.

## Author contributions

AW performed the study, analyzed the data, and wrote the manuscript; YZ, YL, YG, and JC performed the study, and YL also analyzed the data; WL, XL, and XY designed and performed the study. All authors contributed to the article and approved the submitted version.
