# Supplementary material for: High loading-dose of dupilumab resulted in rapid disease control in pediatric patients with atopic dermatitis
Source: Front Immunol. 2023 Apr 20;14:1160710. doi: 10.3389/fimmu.2023.1160710 (PMC10157040; doi:10.3389/fimmu.2023.1160710)
Supplement: Supplementary file 1 [file DataSheet_1.docx]

**
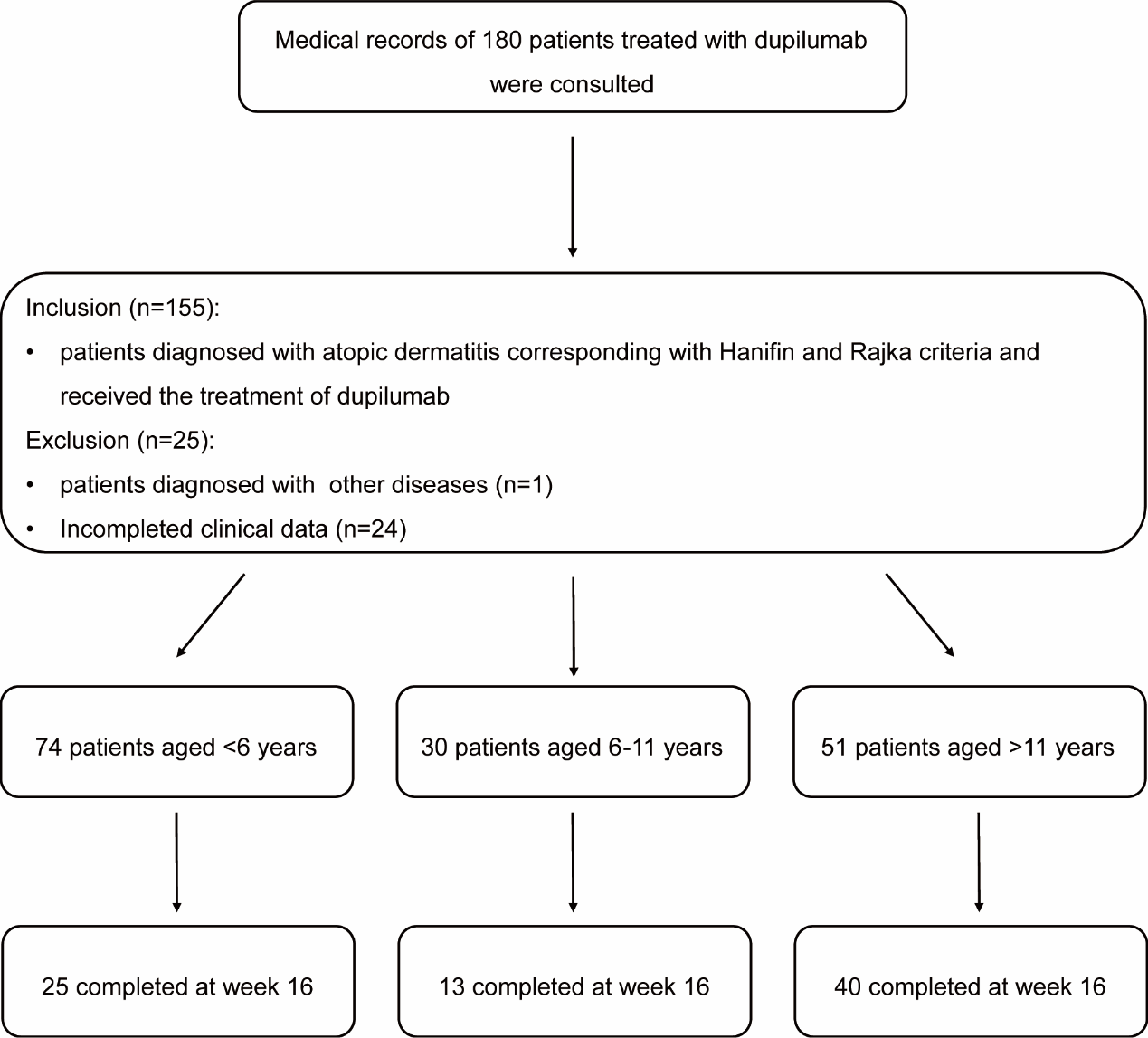
**

**Figure S1.** Study flowchart.

**
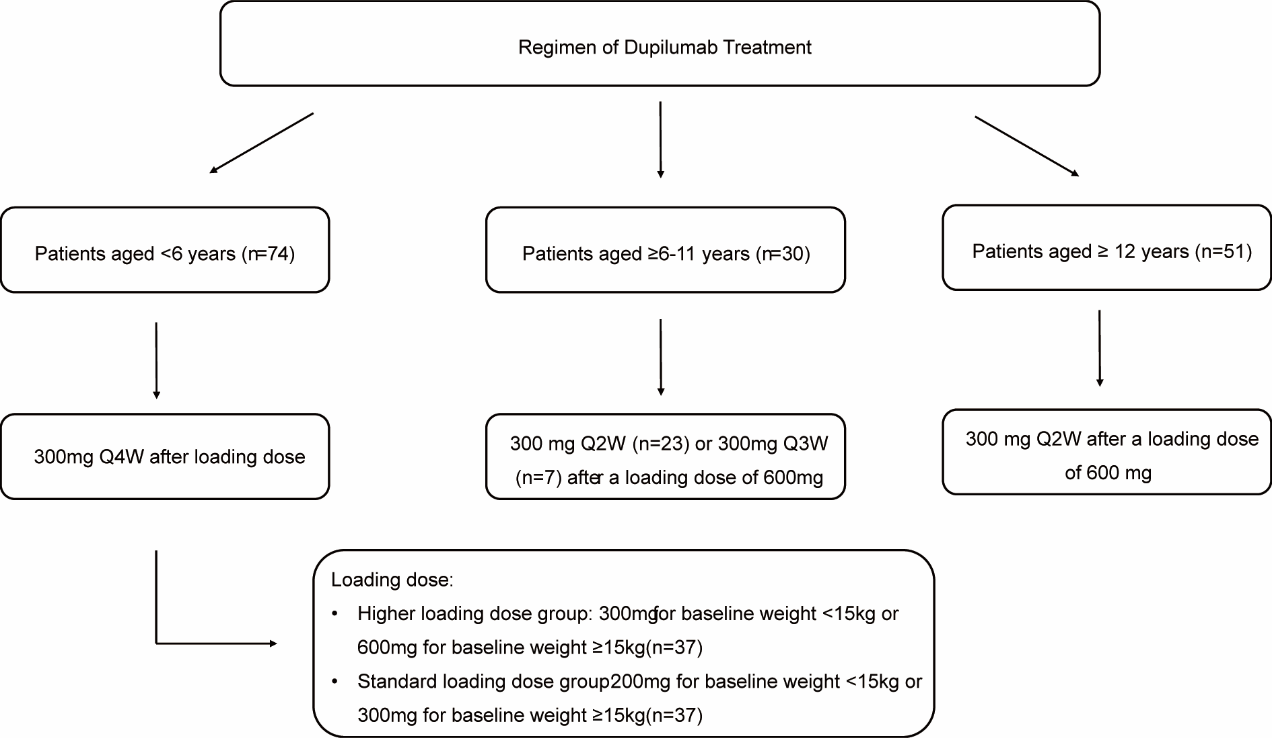
**

**Figure S2.** Regimen of dupilumab treatment.

**
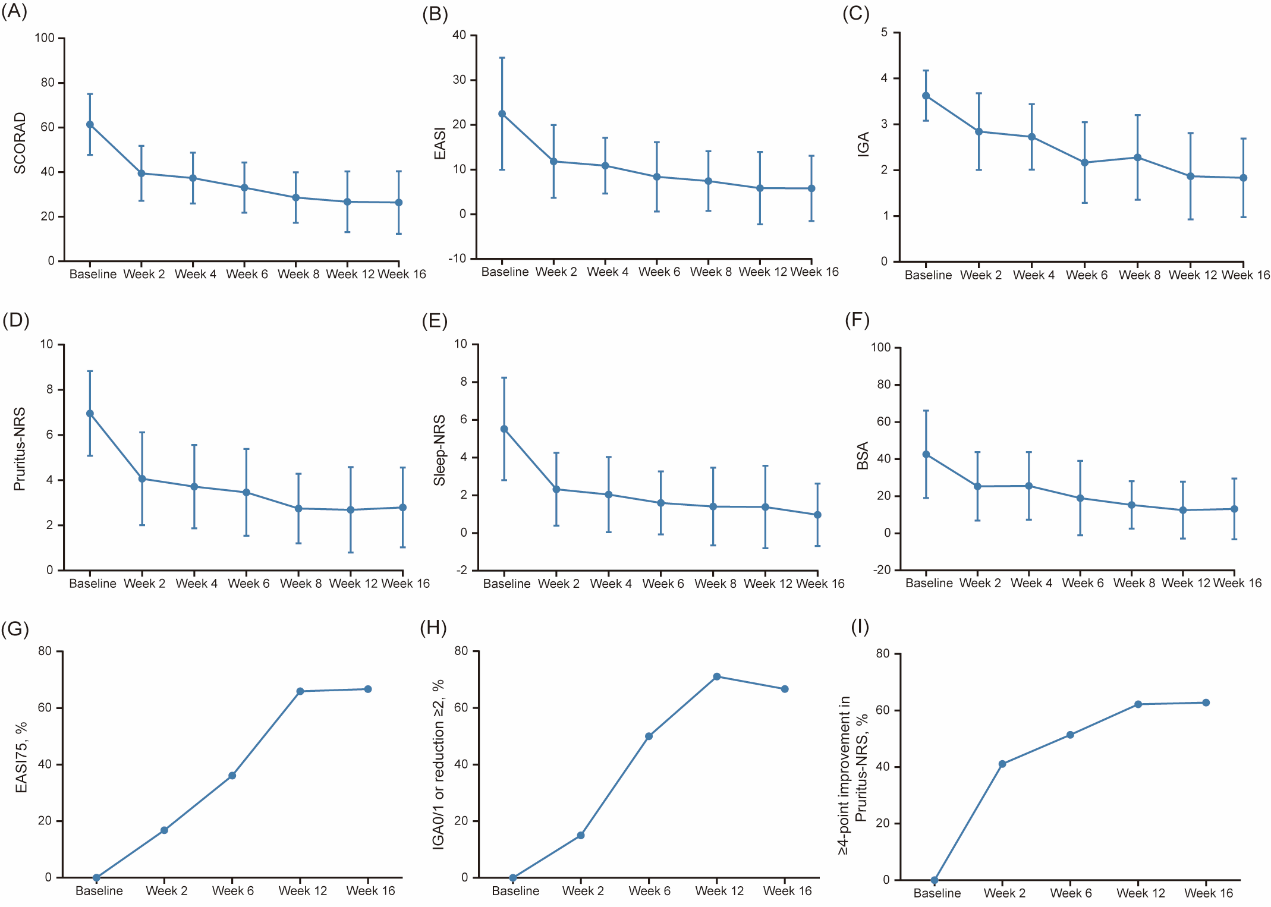
**

**Figure S3.** (A-F) Dynamic change in SCORAD, EASI, IGA, Pruritus-NRS, Sleep-NRS, and BSA during the treatment duration for all patients. Data are shown with mean±SD. (G-I) Dynamic change in percentage of EASI75, IGA0/1 or reduction ≥ 2-point, and ≥4-point improvement in Pruritus-NRS during the treatment duration for all patients.

BSA, body surface area; EASI, Eczema Area and Severity Index; EASI-75, at least 75% improvement from baseline in EASI; IGA, Investigator’s Global Assessment; NRS, Numerical Rating Scale; SCORAD, Scoring Atopic Dermatitis; SD, standard deviation.

**
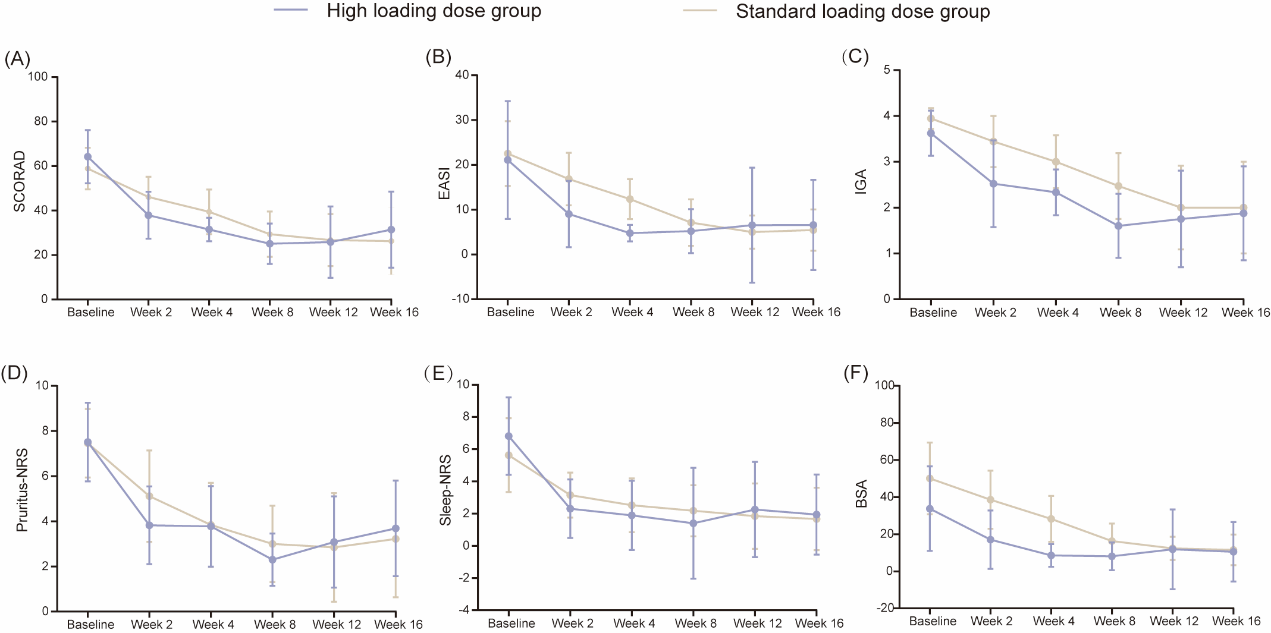
**

**Figure S4.** (A-F) Dynamic change in SCORAD, EASI, IGA, Pruritus-NRS, Sleep-NRS, and BSA between patients receiving high loading dose group and standard loading dose group during the treatment duration. Data are shown with mean±SD.

BSA, body surface area; EASI, Eczema Area and Severity Index; IGA, Investigator’s Global Assessment; NRS, Numerical Rating Scale; SCORAD, Scoring Atopic Dermatitis; SD, standard deviation.

**
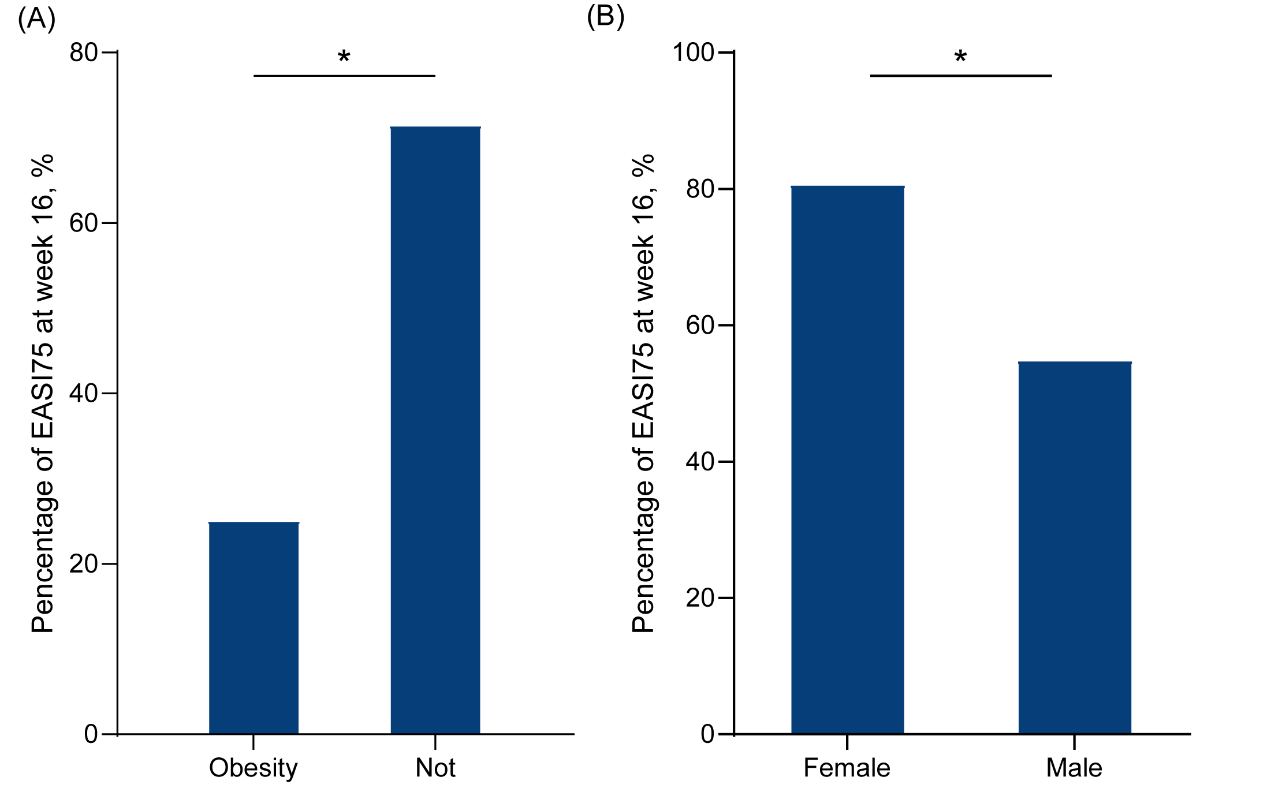
**

**Figure S5.** (A) Comparison of the percentage of EASI75 at week 16 between obese and non-obese patients. (B) Comparison of the percentage of EASI75 at week 16 between female and male patients. **P* < 0.05. EASI, Eczema Area and Severity Index; EASI-75, at least 75% improvement from baseline in EASI.

*
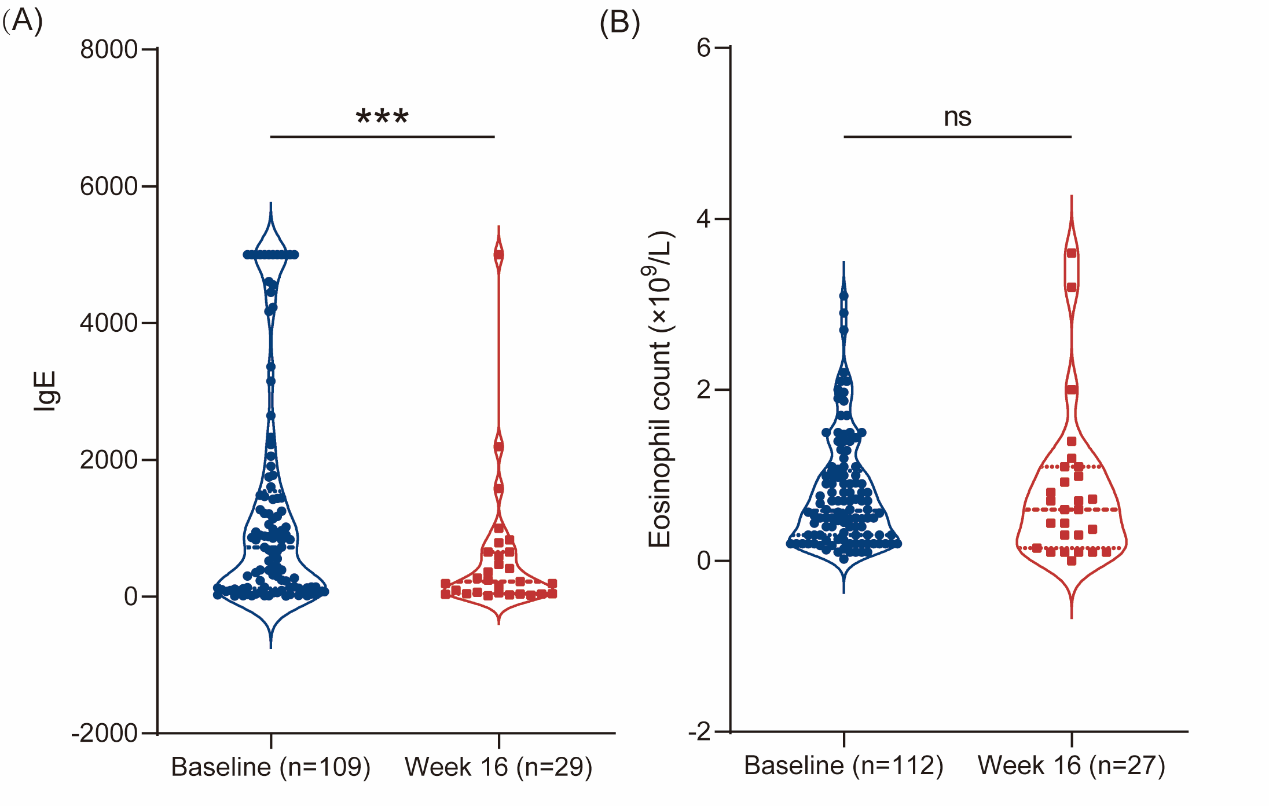
*

**Figure S6.** (A) Change of IgE from baseline to week 16. (B) Change of eosinophil count from baseline to week 16. IgE, Immunoglobulin E. ****P* < 0.001.

**Table S1. Demographic data and clinical characteristics of study population.**

| **Characteristics** | **Total (n=155)** |
| --- | --- |
| Sex: Female, n (%) | 59 (38.1%) |
| Ages (year), mean±SD | 11.8±13.4 |
| Disease duration (years), mean±SD | 7.5±7.3 |
| Baseline BMI, mean±SD  Obesity | 18.0±4.2  16 (10.3%) |
| Baseline SCORAD, mean±SD  > 50, severe, n, %  ≤ 50 and > 25, moderate, n, % | 61.4±13.7  125 (80.6%)  30 (19.4%) |
| Baseline EASI, mean±SD | 22.5±12.6 |
| Baseline IGA, mean±SD | 3.6±0.5 |
| Baseline Sleep-NRS, mean±SD | 5.5±2.7 |
| Baseline Pruritus-NRS, mean±SD | 7.0±1.9 |
| Baseline BSA, mean±SD | 42.6±23.6 |
| Baseline IgE, kU/L  Available for patients, n, % | 1324.1±1660.4  109 (70.3%) |
| Specific allergen available for patients, n, %  Dust mite allergen, n, % | 117 (75.5%)  82 (70.1%) |
| Baseline eosinophil count (×10^9^/L)  Available for patients, n, % | 0.9±1.5  112 (72.3%) |
| Atopic family history | 86 (55.5%) |
| Allergic comorbidities, n, %  Allergic rhinitis, n, %  Asthma, n, %  Food allergy, n, %**^¶^** | 94 (60.6%)  61 (39.4%)  18 (11.6%)  51 (35.7%) |
| History of previous treatment  Topical corticosteroids  Topical calcineurin inhibitors  Topical phosphodiesterase 4 inhibitor  Systemic corticosteroids  Oral antihistamines  Leukotrienes receptor antagonist  Immunosuppressants | 131 (84.5%)  87 (56.1%)  5 (3.2%)  16 (10.3%)  110 (71.0%)  6 (3.9%)  7 (4.5%) |

BSA, body surface area; EASI, Eczema Area and Severity Index; IGA, Investigator’s Global Assessment; IgE: immunoglobulin E; NRS, Numerical Rating Scale; SCORAD, Scoring Atopic Dermatitis; SD, standard deviation.

**^¶^** Food allergy was available for 143 patients.

| **Characteristics** | **Standard loading dose group (n=37)** | **High loading dose group (n=37)** | ***P value*** |
| --- | --- | --- | --- |
| Sex: Female, n (%) | 14 (37.8%) | 16 (43.2%) | 0.81 |
| Ages (year), mean±SD, (range) | 3.0±1.3 | 3.7±1.2 | 0.02 |
| Disease duration (years), mean±SD | 2.7±1.3 | 3.5±1.2 | 0.009 |
| Baseline BMI, mean±SD  Obesity | 15.4±1.8  2 (5.4%) | 15.5±1.7  3 (8.1%) | 0.53  1.00 |
| Baseline SCORAD, mean±SD  > 50, severe, n, %  ≤ 50 and > 25, moderate, n, % | 58.9±9.4  30 (81.1%)  7 (18.9%) | 64.2±12.0  33 (89.2%)  4 (10.8%) | 0.03  0.52 |
| Baseline EASI, mean±SD | 22.5±7.2 | 21.1±13.2 | 0.15 |
| Baseline IGA, mean±SD | 3.9±0.2 | 3.6±0.5 | 0.001 |
| Baseline Sleep-NRS, mean±SD | 5.6±2.3 | 6.8±2.4 | 0.02 |
| Baseline Pruritus-NRS, mean±SD | 7.5±1.5 | 7.5±1.7 | 0.35 |
| Baseline BSA, mean±SD | 50.1±19.3 | 33.8±22.8 | 0.001 |

**Table S2. Clinical features between patients receiving high loading dose group and standard loading dose group at baseline.**

**Table S3. Outcomes between patients receiving high loading dose group and standard loading dose group at week 16.**

| **Outcome** | **Standard loading dose group (n=9)** | **High loading dose group (n=16)** | ***P value*^¶^** |
| --- | --- | --- | --- |
| EASI50 at week 16, n, % | 7 (77.8%) | 13 (81.3%) | 1.00 |
| EASI75 at week 16, n, % | 5 (55.6%) | 12 (75.0%) | 0.39 |
| EASI90 at week 16, n, % | 1 (11.1%) | 6 (37.5%) | 0.35 |
| IGA0/1 or reduction ≥ 2-point at week 16, n, % | 5 (55.6%) | 11 (68.8%) | 0.67 |
| Proportion of ≥4-point improvement in Pruritus-NRS from baseline to week 16, n, % | 5 (55.6%) | 11 (68.8%) | 0.67 |
| SCORAD at week 16, mean±SD | 26.2±15.0 | 32.1±18.0 | N/A |
| Mean percent change in SCORAD from baseline to week 16, mean±SD | −51.2±30.1 | −53.7±25.5 | 0.85 |
| EASI score at week 16, mean±SD | 5.4±4.6 | 6.6±10.1 | N/A |
| Mean percent change in EASI from baseline to week 16, mean±SD | −70.4±25.7 | −73.8±31.4 | 0.69 |

**Table S3. Outcomes between** **patients receiving high loading dose group and standard loading dose group at week 16 (continued).**

| **Outcome** | **Standard loading dose group (n=9)** | **High loading dose group (n=16)** | ***P value*^¶^** |
| --- | --- | --- | --- |
| Pruritus-NRS score at week 16, mean±SD | 3.2±2.6 | 3.7±2.1 | N/A |
| Mean percent change in Pruritus-NRS from baseline to week 16, mean±SD | −49.8±37.0 | −49.2±33.5 | 0.75 |
| Sleep-NRS score at week 16, mean±SD | 1.7±1.9 | 1.9±2.5 | N/A |
| Mean percent change in Sleep-NRS from baseline to week 16, mean±SD | −62.3±48.4 | −71.8±40.5 | 0.56 |
| Percentage of BSA at week 16, mean±SD | 11.6±8.3 | 10.5±16.0 | N/A |
| Mean percent change in the percentage of BSA from baseline to week 16, mean±SD | −61.9±31.2 | −73.5±31.2 | 0.39 |

BSA, body surface area; EASI, Eczema Area and Severity Index; EASI50, at least 50% improvement from baseline in EASI; EASI-75, at least 75% improvement from baseline in EASI; EASI-90, at least 90% improvement from baseline in EASI; IGA, Investigator’s Global Assessment; N/A: not applicable; NRS, Numerical Rating Scale; SCORAD, Scoring Atopic Dermatitis; SD, standard deviation.

**¶ Comparisons between groups.**

**Table S4. Clinical scores and serum levels of biomarkers at baseline and after dupilumab treatment in patients aged <18 years.**

| **Clinical scores** | **Baseline (n=34)** | **After (n=33)** | ***P value*** |
| --- | --- | --- | --- |
| SCORAD, mean±SD | 62.6±14.3 | 29.6±11.9 | <0.001 |
| EASI, mean±SD | 22.4±12.3 | 6.5±7.9 | <0.001 |
| Pruritus-NRS, mean±SD | 7.0±2.0 | 2.6±1.7 | <0.001 |
| **Serum biomarkers (pg/ml)** | **Baseline (n=34)** | **After (n=34)** | ***P value*** |
| IL-10, mean±SD | 8.4±4.4 | 5.9±2.6 | <0.001 |
| CCL18/PARC, mean±SD | 18743.2±6481.9 | 13196.7±4889.9 | <0.001 |
| IL-4, mean±SD | 103.7±22.2 | 93.6±12.9 | <0.001 |
| CCL26, mean±SD | 10.2±5.4 | 9.5±1.4 | 0.68 |
| CD25, mean±SD | 711.3±399.7 | 518.5±204.3 | <0.001 |
| TSLP, mean±SD | 1.1±0.3 | 1.4±0.2 | <0.001 |
| Periostin, mean±SD | 132606.8±40294.3 | 122957.1±41204.6 | 0.20 |
| IL-12 p70, mean±SD | 56.1±14.6 | 52.6±15.3 | 0.39 |
| CCL17/TARC, mean±SD | 4707.7±7386.2 | 661.2±651.8 | <0.001 |
| IL-21, mean±SD | 28.1±42.8 | 20.8±15.1 | 0.003 |
| CCL11, mean±SD | 62.9±19.2 | 56.2±21.2 | 0.13 |

**Table S4. Clinical scores and serum levels of biomarkers at baseline and after dupilumab treatment in patients aged <18 years (continued).**

| **Serum biomarkers (pg/ml)** | **Baseline (n=34)** | **After (n=34)** | ***P value*** |
| --- | --- | --- | --- |
| IL-18, mean±SD | 238.4±142.7 | 172.4±177.8 | <0.001 |
| TNF-α, mean±SD | 5.2±2.8 | 4.9±3.7 | 0.03 |
| IL-36β, mean±SD | 0.9±0.8 | 0.6±0.8 | <0.001 |
| IL-1β, mean±SD | 2.1±9.2 | 0.2±0.5 | <0.001 |
| IL-5, mean±SD | 0.7±0.9 | 0.3±0.5 | 0.03 |
| IL-6, mean±SD | 1.2±1.2 | 0.8±1.9 | <0.001 |
| IFN-γ, mean±SD | 1.3±1.2 | 1.3±1.0 | 0.98 |
| IL-17A, mean±SD | 0.5±0.5 | 0.3±0.4 | 0.006 |

CCL, C-C motif ligand; EASI, Eczema Area and Severity Index; IFN, interferon; IL, interleukin; NRS, Numerical Rating Scale; PARC, pulmonary and activation-regulated chemokine; SCORAD, Scoring Atopic Dermatitis; SD, standard deviation; TARC, thymus and activation-regulated chemokine; TNF, tumor necrosis factor; TSLP, thymic stromal lymphopoietin.

**Table S5. Adverse events during dupilumab treatment between high loading dose group and standard loading dose group at week 16.**

| **Adverse events** | **Standard loading dose (n=37)** | **High loading dose (n=37)** | ***P value*^¶^** |
| --- | --- | --- | --- |
| Conjunctivitis or aggravation of conjunctivitis | 4 (10.8%) | 4 (10.8%) | 1.00 |
| Facial erythema | 0 | 3 (8.1%) | 0.24 |
| Injection-site reaction | 0 | 0 | 1.00 |
| Flu-like symptoms | 1 (2.7%) | 1 (2.7%) | 1.00 |
| Joint pain | 0 | 0 | 1.00 |
| Herpes simplex virus infection | 1 (2.7%) | 0 | 1.00 |
| Drowsiness | 1 (2.7%) | 0 | 1.00 |

**Table S6. Change in the use of topical corticosteroids at baseline and week 16.**

| **Topical corticosteroids** | **Baseline (n=155)** | **Week 16 (n=78)** | ***P value*** |
| --- | --- | --- | --- |
| Very-high-potency corticosteroids, n, % | 27 (17.4%) | 9 (11.5%) | 0.34 |
| High-potency corticosteroids, n, % | 75 (48.4%) | 10 (12.8%) | <0.001 |
| Medium-potency corticosteroids, n, % | 32 (20.6%) | 22 (28.2%) | 0.25 |
| Low-potency corticosteroids, n, % | 12 (7.7%) | 2 (2.6%) | 0.15 |
